# Mitochondria-Associated Endoplasmic Reticulum Membrane Biomarkers in Coronary Heart Disease and Atherosclerosis: A Transcriptomic and Mendelian Randomization Study

**DOI:** 10.3390/cimb48010075

**Published:** 2026-01-12

**Authors:** Junyan Zhang, Ran Zhang, Li Rao, Chenyu Tian, Shuangliang Ma, Chen Li, Yong He, Zhongxiu Chen

**Affiliations:** 1 Department of Cardiology, West China Hospital of Sichuan University, Chengdu 610041, China; zhangjunyan1@stu.scu.edu.cn (J.Z.); 17882085081@163.com (R.Z.); lrlz1989@163.com (L.R.); msl15388777340@163.com (S.M.); lichen8374@wchscu.cn (C.L.); heyongmd@wchscu.cn (Y.H.); 2 West China Biomedical Big Data Center, West China Hospital of Sichuan University, Chengdu 610041, China; tianchenyu@wchscu.cn

**Keywords:** Mitochondria-associated endoplasmic reticulum membranes (MAMs), mendelian randomization, coronary heart disease, machine learning, diagnostic model

## Abstract

Background: Coronary heart disease (CHD) remains a leading cause of morbidity and mortality worldwide. Mitochondria-associated endoplasmic reticulum membranes (MAMs) have recently emerged as critical mediators in cardiovascular pathophysiology; however, their specific contributions to CHD pathogenesis remain largely unexplored. Objective: This study aimed to identify and validate MAM-related biomarkers in CHD through integrated analysis of transcriptomic sequencing data and Mendelian randomization, and to elucidate their underlying mechanisms. Methods: We analyzed two gene expression microarray datasets (GSE113079 and GSE42148) and one genome-wide association study (GWAS) dataset (ukb-d-I9_CHD) to identify differentially expressed genes (DEGs) associated with CHD. MAM-related DEGs were filtered using weighted gene co-expression network analysis (WGCNA). Functional enrichment analysis, Mendelian randomization, and machine learning algorithms were employed to identify biomarkers with direct causal relationships to CHD. A diagnostic model was constructed to evaluate the clinical utility of the identified biomarkers. Additionally, we validated the two hub genes in peripheral blood samples from CHD patients and normal controls, as well as in aortic tissue samples from a low-density lipoprotein receptor-deficient (LDLR−/−) atherosclerosis mouse model. Results: We identified 4174 DEGs, from which 3326 MAM-related DEGs (DE-MRGs) were further filtered. Mendelian randomization analysis coupled with machine learning identified two biomarkers, DHX36 and GPR68, demonstrating direct causal relationships with CHD. These biomarkers exhibited excellent diagnostic performance with areas under the receiver operating characteristic (ROC) curve exceeding 0.9. A molecular interaction network was constructed to reveal the biological pathways and molecular mechanisms involving these biomarkers. Furthermore, validation using peripheral blood from CHD patients and aortic tissues from the Ldlr−/− atherosclerosis mouse model corroborated these findings. Conclusions: This study provides evidence supporting a mechanistic link between MAM dysfunction and CHD pathogenesis, identifying candidate biomarkers that have the potential to serve as diagnostic tools and therapeutic targets for CHD. While the validated biomarkers offer valuable insights into the molecular pathways underlying disease development, additional studies are needed to confirm their clinical relevance and therapeutic potential in larger, independent cohorts.

## 1. Introduction

Coronary heart disease (CHD), a complex and multifactorial cardiovascular condition, stands as a predominant cause of global morbidity and mortality [1]. The pathophysiology of CHD is intricate, involving genetic predispositions, environmental factors, and lifestyle choices that contribute to the development and progression of the disease [2,3]. Despite advancements in therapeutic strategies and preventive measures, CHD continues to impose a significant burden on healthcare systems, underscoring the need for a deeper understanding of its underlying mechanisms and the identification of novel biomarkers for early diagnosis and targeted treatment.

Mitochondria-associated endoplasmic reticulum membranes (MAMs) are specialized membrane microdomains that form critical contact sites between the endoplasmic reticulum (ER) and mitochondria [4,5]. These structures are essential for maintaining cellular homeostasis by facilitating the exchange of lipids, calcium, and other metabolites between the two organelles [4,6]. MAMs are also implicated in the regulation of cellular signaling, mitochondrial dynamics, autophagy, and apoptosis, which are processes that are disrupted in various pathological conditions, including cardiovascular diseases [7,8,9].

**Figure 1:**
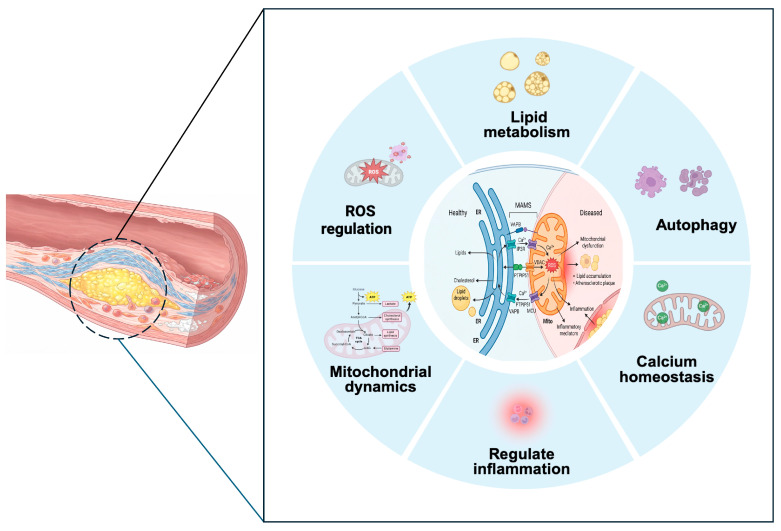
Potential functions and mechanisms of MAMs in the pathogenesis of atherosclerosis. Adapted from refs. [10,11].

The role of MAMs in cardiovascular health and disease is an emerging area of research. Recent studies suggest that MAMs may play a role in the pathogenesis of CHD by influencing cellular responses to stress, modulating lipid metabolism, and affecting the inflammatory response [10,11] (Figure 1). However, the specific mechanisms by which MAMs contribute to CHD and their potential as therapeutic targets remain to be fully elucidated.

In this context, the present study aims to identify and validate MAM-related biomarkers in CHD using transcriptomic sequencing data combined with Mendelian randomization analysis and to explore their underlying mechanisms.

## 2. Materials and Methods

### 2.1. Data Collection

In the present study, we accessed two gene expression microarray datasets, GSE113079 and GSE42148, from the Gene Expression Omnibus (GEO) database. GSE113079 contains 93 samples from individuals with Coronary Heart Disease (CHD) and 48 samples from normal control Peripheral Blood Mononuclear Cells (PBMCs). In contrast, GSE42148 comprises 13 samples from CHD patients and 11 samples from normal control PBMCs. Additionally, we procured a Genome-Wide Association Study (GWAS) dataset, ukb-d-I9_CHD, from the IEU OpenGWAS database, which includes 10,157 samples from CHD cases and 351,037 samples from control individuals. Moreover, we conducted a literature review to identify a total of 28 genes associated with the MAMs.

### 2.2. Data Quality Control and Differential Expression Analysis

In this project, we utilized the GSE113079 dataset as our training set and performed differential expression analysis using the “limma” package (version 3.52.4). Prior to this analysis, to ensure the exclusion of batch effects between samples within the training set, we initially conducted principal component analysis (PCA) as part of our quality control measures. Samples that fell outside the 95% confidence interval were deemed as outliers and were subsequently removed from the dataset. Following the quality control step, we proceeded with the differential expression analysis, retaining genes with an absolute value of log2 Fold Change (log2FC) greater than 0.5 and a *p*-value less than 0.05. These genes were defined as differentially expressed genes (DEGs). To visually represent the results, we employed the “ggplot2” package (version 3.3.6) to generate volcano plots and the “pheatmap” package (version 1.0.12) to create heatmaps, which facilitated the interpretation and visualization of the DEGs identified in our analysis.

### 2.3. Identification of Differentially Expressed MAM-Related Genes

To incorporate the mechanisms associated with the MAM into our study, we initially employed the “GSVA” package (version 1.48.3) with the ssGSEA method, utilizing a gene set related to MAM related genes (MRGs) as the background, to calculate MAM-related scores for each sample in the training set. We then compared the score differences between the CHD and Control groups using the “wilcoxon” test. Subsequently, we performed weighted gene co-expression network analysis (WGCNA) using the “WGCNA” package (version 1.72.1). In this process, we first identified and removed outliers from the training set. We then constructed a topological network and calculated the signed R^2^ to assess the degree to which the network approximated a scale-free network. The soft threshold at which the R^2^ first reached 0.85 was selected as the optimal soft threshold for subsequent co-expression network construction, and all genes were assigned to different modules. Based on the “pearson” correlation analysis between the modules and the MAM-related scores, we identified the module most closely associated with the scores and defined the genes within this module as hub genes. Ultimately, we used the “ggvenn” package (version 1.2.2) to find the intersection of hub genes and DEGs, and we defined these genes as MAM-related differentially expressed genes (DE-MRGs).

### 2.4. Functional Enrichment Analysis Based on DE-MRGs

In an effort to gain initial insights into the biological processes and molecular mechanisms that DE-MRGs may participate in, this investigation leveraged the “clusterProfiler” R package (version 4.7.1.001) to execute a functional enrichment analysis of these genes. The analysis was conducted utilizing data from the Gene Ontology (GO) and the Kyoto Encyclopedia of Genes and Genomes (KEGG) databases. The GO database was further divided into three aspects: Cellular Components (CC), Molecular Functions (MF), and Biological Process (BP). We retained all results with *p*-values less than 0.05 for further analysis.

### 2.5. Identification of MAM-Related Biomarkers in CHD

In our methodology, we leveraged the “TwoSampleMR” R package (version 0.5.7) to execute Mendelian randomization analysis focusing on DE-MRGs. This analysis aimed to filter and identify genes with a putative direct causal relationship with CHD [12]. Within the ukb-d-I9_CHD dataset, we performed association analysis to select instrumental variable single nucleotide polymorphisms (SNPs) with a significance threshold of *p* < 5 × 10^−8^. Subsequently, we deployed the Inverse variance weighted (IVW), MR Egger, and Weighted median algorithms for Mendelian randomization analysis, retaining genes with IVW-*p* values less than 0.05 as prospective candidate genes. We proceeded by constructing a protein–protein interaction network utilizing the STRING database and “Cytoscape” software (version 3.8.2) [13]. Each candidate gene was scored within the network using the Betweenness and Stress algorithms through the “cytoHubba” plugin. We then identified the intersection of the top 20 genes with the highest scores from each algorithm to further refine our candidate list. Next, we employed the LASSO machine learning algorithm from the “glmnet” package (version 4.1.7), the SVM-RFE algorithm from the “e1071” package (version 1.7.13), and the Boruta algorithm from the “Boruta” package (version 8.0.0) to concurrently screen these genes [14]. Only those genes identified by all three algorithms were retained, and their expression levels were concurrently validated. Finally, we conducted a series of validation tests on the remaining candidate genes. This included the Steiger’s direction test to confirm the correct direction of analysis, a heterogeneity test (assuming SNPs to be randomly distributed according to Mendel’s second law if the Q value was greater than 0.05), a pleiotropy test (assuming SNPs to lack horizontal pleiotropy if the *p* value was greater than 0.05), and a leave-one-out test. Genes that passed all these tests were retained and designated as biomarkers.

### 2.6. Construction of a Diagnostic Model Based on Biomarkers

To further explore the diagnostic value of the identified biomarkers, we constructed a CHD diagnostic model using the “rms” package (version 6.7.1) [15]. The model’s goodness-of-fit was assessed using the Hosmer–Lemeshow test, with a *p*-value greater than 0.05 indicating a well-fitted model. Calibration curves were plotted to validate the diagnostic capacity of the biomarkers. Additionally, we utilized the “pROC” package (version 1.18.4) to generate the receiver operating characteristic (ROC) curves for the model and calculated the area under the curve (AUC) to further verify the model’s predictive ability [16].

### 2.7. In-Depth Exploration of the Molecular Mechanisms of Biomarkers

To further explore the molecular mechanisms of the identified biomarkers, we conducted a multifaceted analysis. Initially, we utilized geneMANIA to construct a molecular interaction network based on the biomarkers, delving into their mechanisms at the molecular interaction level. Subsequently, we employed the “psych” package (version 2.3.6) to perform Spearman correlation analysis between the biomarkers and all genes, ranking the results by correlation magnitude. We then used the “clusterProfiler” with the CP: KEGG gene sets from the MsigDB database as the background gene set, conducting single-gene GSEA enrichment analysis and retaining all results with Padj less than 0.05. Additionally, we applied the CIBERSORT algorithm to calculate the proportions of 22 immune-infiltrating cells across all samples and used the “wilcoxon” test to compare the proportion differences between the CHD and Control groups. We also explored the correlation between biomarkers and cells with significant intergroup differences using Spearman correlation analysis. Finally, we predicted miRNAs with potential regulatory effects on the biomarkers through the miRDB database and identified potential lncRNA regulators of these miRNAs using the starBase database, ultimately constructing a non-coding regulatory network centered on the biomarkers. We also used Networkanalyst for the prediction of transcription factors potentially regulating the biomarkers and constructed a transcription factor regulatory network.

### 2.8. Quantitative Real-Time Polymerase Chain Reaction (qRT-PCR)

In total, 10 matching blood samples were collected from healthy subjects and CHD patients in the West China Hospital of Sichuan University. Patients with CHD were selected for undergoing invasive coronary angiography for a diagnosis of CHD and were matched with the control group based on gender, age, general condition, and comorbidities. Total RNA was isolated from blood samples using Trizol reagent (Thermo Fisher Scientific, Beijing, China), and its concentration was determined after dissolving in DEPC-treated water. The RNA was then reverse transcribed into cDNA using the SureScript First-Strand cDNA Synthesis Kit (Servicebio, Wuhan, China). Subsequently, qPCR was conducted using the CFX96 real-time quantitative PCR instrument (Bio-Rad, Shanghai, China) using the following protocol: 95 °C for 1 min for pre-denaturation; 95 °C for 20 s for denaturation; 55 °C for 20 s for annealing; and 72 °C for 30 s for extension, repeating the first three steps for a total of 40 cycles. GAPDH served as the internal reference gene, and the relative expression levels of the target genes were calculated using the 2^−ΔΔCt^ method. All analyses were performed in triplicate. The primer sequences are listed in Table 1.

### 2.9. Development of Animal Models for Atherosclerosis

All animal procedures were conducted in strict accordance with the Guide for the Care and Use of Laboratory Animals and approved by the Animal Ethics Committee of West China Hospital, Sichuan University (Protocol No. 20230423003).

Ten male LDLR−/− mice (6 weeks old, 18–20 g) were obtained from Gempharmatech Co., Ltd. (Nanjing, China) and were housed in the specific pathogen-free facility at the Laboratory Animal Center of West China Hospital, Sichuan University. The animals were maintained under standard conditions with free access to food and water, at 24 °C ambient temperature, 50–60% relative humidity, and a 12 h light/12 h dark cycle.

The mice were randomly assigned into two groups (*n* = 5 per group): the high-fat diet (HFD) group received a high-fat diet (D12108C, Research Diets, New Brunswick, NJ, USA; containing 40% kcal from fat, 43% from carbohydrates, and 17% from protein) for 12 weeks, while the normal chow diet (NCD) group was fed standard chow (containing approximately 10% kcal from fat, 70% from carbohydrates, and 20% from protein) for 12 weeks. At the conclusion of the feeding period, mice were euthanized by CO_2_ inhalation followed by cervical dislocation and arterial tissues were collected for subsequent analysis.

### 2.10. Oil Red O Staining

For aortic oil red O staining, intact aortae spanning from the aortic arch to the iliac bifurcation were harvested and meticulously cleared of surrounding adipose tissue. Following 24 h fixation in 4% paraformaldehyde and two PBS rinses, vessels were opened longitudinally. The exposed aortic intima was then incubated in oil red O solution at 37 °C for 60 min. Subsequent differentiation with 75% ethanol revealed lipid-laden plaques as orange-red to bright red deposits against a colorless background. After two distilled water washes, specimens were digitally photographed.

### 2.11. IHC Staining

Sectioned specimens first underwent solvent-based wax removal and progressive tissue rehydration. Antigen accessibility was enhanced via thermal processing in sodium citrate solution (0.1 M) at 94 °C for twenty minutes then allowed to cool naturally. To neutralize intrinsic peroxidase activity, slides received 3% H_2_O_2_ treatment for 25 min while protected from light. A half-hour preincubation with 3% BSA minimized background staining before applying primary antibodies (anti-GPR68 and anti-DHX36, ABclonal, Woburn, MA, USA) and refrigerating overnight. Post-washing, sections were treated with enzyme-linked secondary antibody (goat anti-rabbit IgG-HRP, ABclonal) for ninety minutes. Immunocomplexes were detected using diaminobenzidine substrate, with cell nuclei visualized by hematoxylin staining. An Olympus BX63 (Olympus, Tokyo, Japan) microscopy system captured representative fields.

### 2.12. Immunofluorescence Staining

Atherosclerotic lesion sections from the aortic root of LDLR−/− mice underwent paraformaldehyde fixation (4%) and were then embedded in optimal cutting temperature compound. Eight-micrometer frozen sections were generated using Leica cryostat technology (Leica, Wetzlar, Germany). Following 60 min desiccation at ambient temperature, specimens were rehydrated in PBS (1×, 5 min). Epitope unmasking employed thermal sodium citrate treatment (Proteintech PR30001, Wuhan, China) with subsequent triple buffer washing. Permeabilization and blocking proceeded simultaneously using PBS containing 1% BSA and 0.25% Triton X-100 (1 h, room temperature). Overnight refrigerated incubation (4 °C) with anti-GPR68 and anti-DHX36 primary antibodies (ABclonal) preceded fluorescent secondary antibody application (30 min). Nuclear visualization via DAPI staining (5 min) concluded the protocol after three PBS rinses. Image acquisition employed Olympus BX63 microscopy.

## 3. Results

### 3.1. Identification of 4174 Differentially Expressed Genes

**Figure 2:**
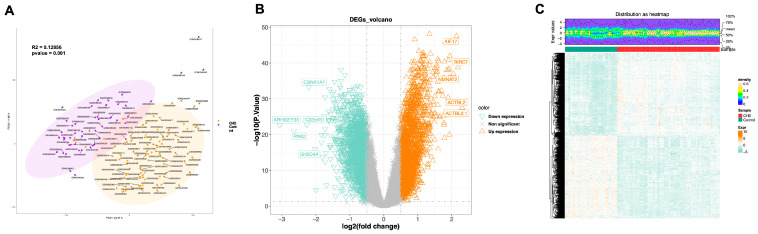
Differential expression analysis. (**A**) PCA of samples. The *x*-axis and *y*-axis represent Principal Component 1 and Principal Component 2, respectively, with the percentages indicating the variance explained by each principal component. The shaded area represents the 95% confidence interval. (**B**) Volcano plot of differentially expressed genes. The orange upward triangles represent upregulated differentially expressed genes, while the green downward triangles represent downregulated differentially expressed genes. The gray X marks indicate genes that do not have significant statistical significance. (**C**) Heatmap of differentially expressed gene expression levels. The top figure represents the density distribution of differentially expressed gene levels, while the *x*-axis below represents the samples and the *y*-axis represents the genes. The green section at the top indicates Control samples, and the red section indicates CHD samples. In the heatmap, orange represents high expression genes, and green represents low expression genes.

To ascertain the absence of batch effects within the dataset, we conducted a principal component analysis (PCA) on all the samples and detected 16 outliers. Following the exclusion of these outlier samples, the training dataset comprised 83 samples from individuals with CHD and 42 control samples (Figure 2A). Subsequently, in our differential expression analysis, we identified a total of 4174 genes that were differentially expressed, with 2094 genes being significantly upregulated and 2080 genes being significantly downregulated (Figure 2B,C).

### 3.2. Obtaining of 3326 DE-MRGs Through WGCNA

**Figure 3:**
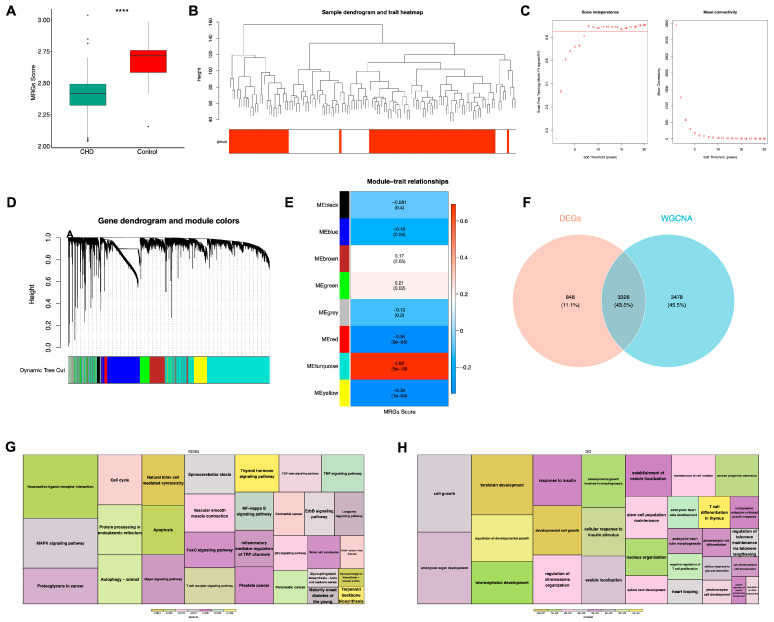
WGCNA Results. (**A**) Differences in MRGs Scores between the CHD and Control groups. **** *p* < 0.0001. (**B**) Sample clustering dendrogram. (**C**) Soft thresholding selection. The *x*-axis in both plots represents the weight parameter power value; the *y*-axis of the left plot shows the scale-free fit index (signed R^2^), where a higher squared correlation coefficient indicates that the network approaches a scale-free distribution. The *y*-axis of the right plot represents the mean of the adjacency functions of all genes in the corresponding gene module. (**D**) Dynamic tree cut. (**E**) Heatmap of module-score correlations. (**F**) Venn diagram of DE-MRGs. (**G**) Hierarchical clustering of DE-MRGs GO enrichment analysis, where box size indicates the number of genes contained, and color represents significance. (**H**) Hierarchical clustering of DE-MRGs KEGG enrichment analysis, where box size indicates the number of genes contained, and color represents significance.

To systematically quantify the activity of MAM-related molecular mechanisms within each sample of the training set, we calculated the MAM-related scores for each sample and observed a significant difference in these scores between the CHD and Control groups, confirming the involvement of MAM-related molecular mechanisms in the pathogenesis and progression of CHD (Figure 3A). Subsequently, we performed WGCNA, using a soft-threshold value of 8 to construct a co-expression network, successfully assigning all genes into 7 distinct modules (Figure 3B–D). Through correlation analysis, we identified the turquoise module as having the highest correlation with the MAM-related scores, thus defining it as the key module, with the 6804 genes within it designated as key genes (Figure 3E). Ultimately, by intersecting the data, we obtained 3326 DE-MRGs (Figure 3F). Furthermore, we conducted functional enrichment analysis based on them and discovered that they were primarily associated with pathways such as “forebrain development,” “telencephalon development,” and “Natural killer cell mediated cytotoxicity” (Figure 3G,H).

### 3.3. Two Biomarkers Were Identified Through Mendelian Randomization and Machine Learning Algorithms

**Figure 4:**
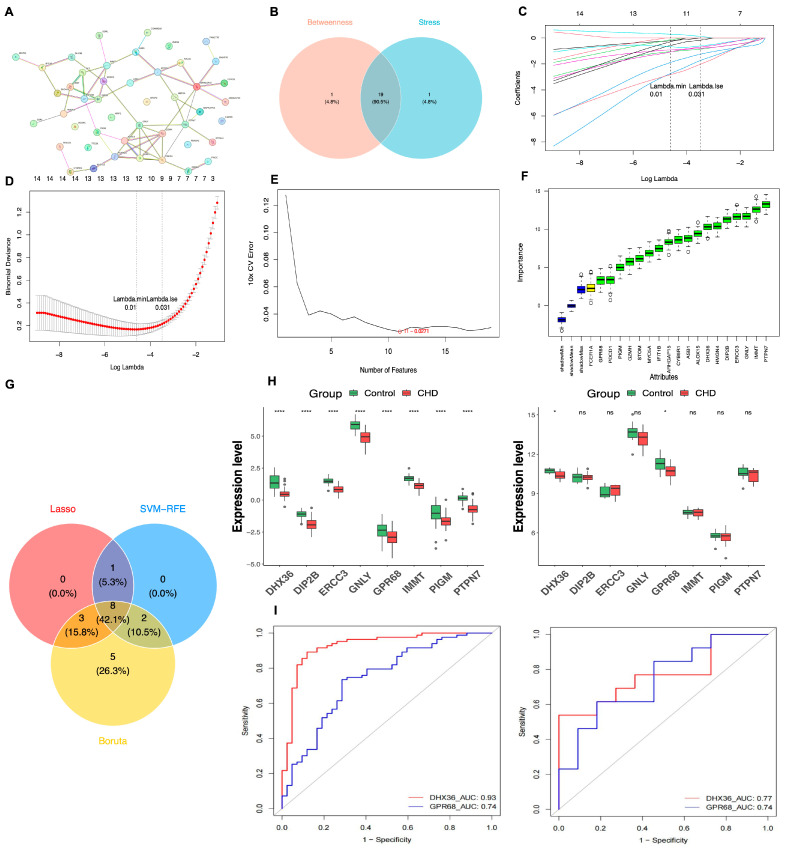
Construction of the PPI network, machine learning, and validation of biomarker expression levels and diagnostic value analysis. (**A**) Potential core gene PPI network. (**B**) Top 20 genes from two algorithms; the left panel shows the Betweenness algorithm, while the right panel displays the Stress algorithm. (**C**) Venn diagram of the two algorithms. (**D**) Lasso regression analysis. (**E**) SVM-RFE analysis; the *x*-axis represents the number of genes, and the *y*-axis represents the error rate. (**F**) Boruta analysis; the *x*-axis shows genes, with red indicating rejected features, green indicating important features, yellow indicating ambiguous important features, and blue representing the minimum, average, and maximum Z values of shadow attributes. (**G**) Venn diagram of three machine learning algorithms. (**H**) Expression levels of intersecting genes; the left panel represents the training set GSE113079, and the right panel represents the validation set GSE42148. The *x*-axis displays the intersecting genes, while the *y*-axis shows expression levels. Box colors indicate sample grouping; significance levels are indicated at the top: ns, not significant; *, *p* < 0.05; ****, *p* < 0.0001. (**I**) ROC curves for biomarkers; the left panel shows the training set GSE113079, and the right panel shows the validation set GSE42148. The *x*-axis represents the false positive rate, where a smaller X indicates higher accuracy; the *y*-axis represents the true positive rate, where a larger Y indicates higher accuracy. By evaluating true positive and false positive rates across different thresholds, a curve can be constructed that extends from the lower left to the upper right and bends towards the upper left; a classifier with no discriminative power between positive and negative classes will form a diagonal line, with endpoints at (0,0) and (1,1).

**Figure 5:**
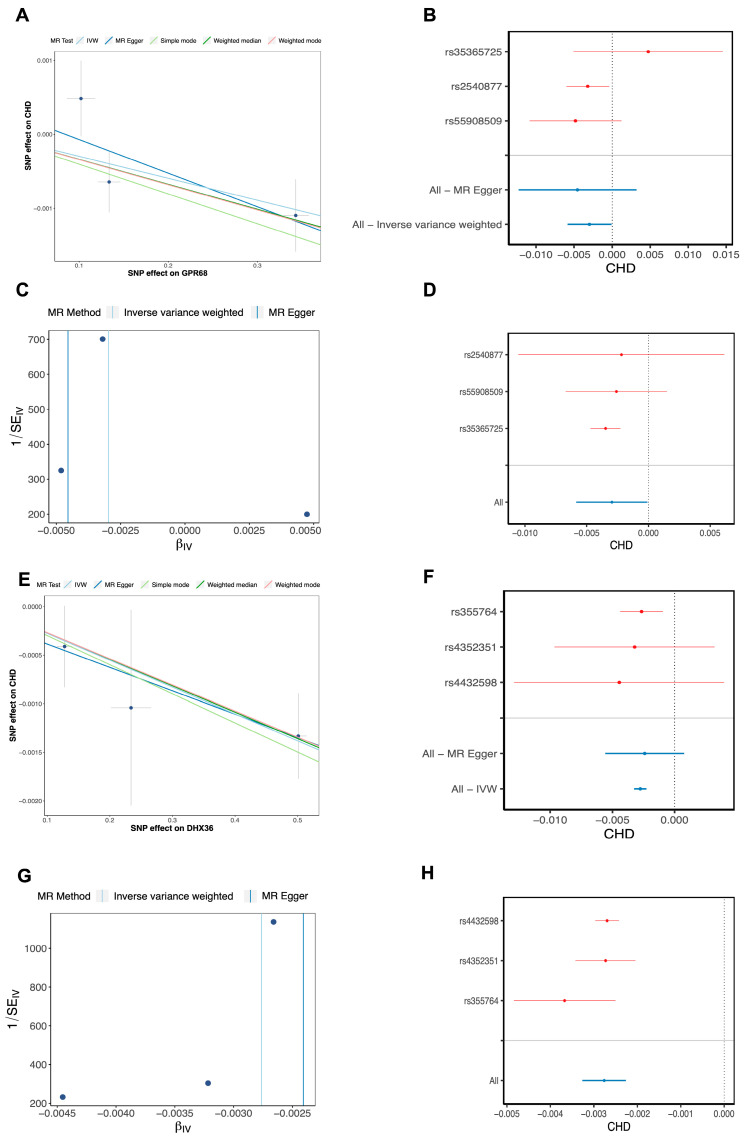
MR analysis results for biomarkers. (**A**) Scatter plot of DHX36 and CHD. (**B**) Forest plot of DHX36 and CHD. (**C**) Funnel plot of DHX36 and CHD. (**D**) Leave-one-out sensitivity analysis for DHX36 and CHD. (**E**) Scatter plot of GPR68 and CHD. (**F**) Forest plot of GPR68 and CHD. (**G**) Funnel plot of GPR68 and CHD. (**H**) Leave-one-out sensitivity analysis for GPR68 and CHD.

Through the application of Mendelian randomization, we successfully identified 46 DE-MRGs that withstood the scrutiny of the IVW algorithm, thereby designating these as candidate genes. Utilizing the Betweenness and Stress algorithms within the PPI network constructed from these genes, we conducted a preliminary screening process, which narrowed down our list to 19 candidate genes. Further refinement was achieved by employing the LASSO, SVM-RFE, and Boruta algorithms, leading to the identification of eight genes: GPR68, PTPN7, DIP2B, DHX36, PIGM, IMMT, ERCC3, and GNLY (Figure 4A–I). Subsequently, these genes underwent a series of rigorous validation tests, including Steiger’s test, heterogeneity test, pleiotropy test, and leave-one-out test, all within the framework of Mendelian randomization analysis. This comprehensive validation process culminated in the identification of two genes, DHX36 and GPR68, which demonstrated a direct causal relationship with CHD and were thus defined as biomarkers (Figure 5A–H) (Supplementry Tables S1–S3). These findings not only enhance our understanding of the genetic underpinnings of CHD but also hold potential for the development of novel therapeutic targets.

### 3.4. Biomarkers Exhibit Good Diagnostic Utility

**Figure 6:**
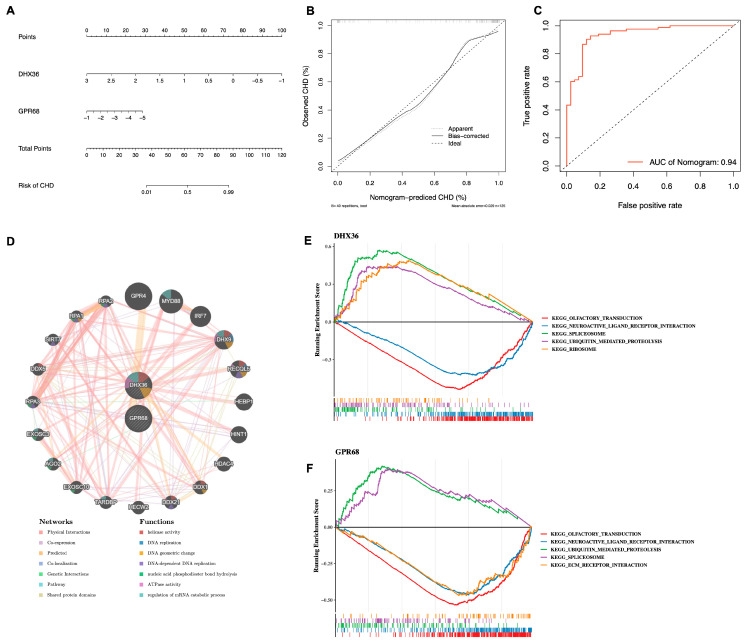
Construction of the nomogram, GGI network, and GSEA enrichment analysis. (**A**) Nomogram for biomarkers. (**B**) Calibration curve for the nomogram. (**C**) ROC curve for the nomogram model. (**D**) GGI network; the color of the connecting lines represents different modes of action, while the color of the circles indicates the functional enrichment of each gene. (**E**,**F**) GSEA enrichment analysis for biomarkers; the line plot shows the Enrichment Score (ES), with the *x*-axis representing the ranked genes and the *y*-axis representing the corresponding Running ES. The peak in the line plot indicates the Enrichment Score for the pathway gene set, with the genes preceding the peak being the core genes of that gene set. The lower part of the plot marks the genes located within that gene set.

In the present study, we successfully developed a diagnostic model for CHD utilizing the identified biomarkers and created an accompanying nomogram (Figure 6A). The calibration curve’s slope was close to 1, signifying a high degree of precision in the model’s predictive capabilities (Figure 6B). Moreover, the area under the ROC curve exceeded 0.9, which not only substantiates the model’s diagnostic efficacy but also underscores the biomarkers’ potent diagnostic potential. These results collectively suggest that our model offers a reliable and robust tool for CHD diagnosis (Figure 6C).

### 3.5. Biological Markers Participate in Ubiquitin-Mediated Proteolysis

**Figure 7:**
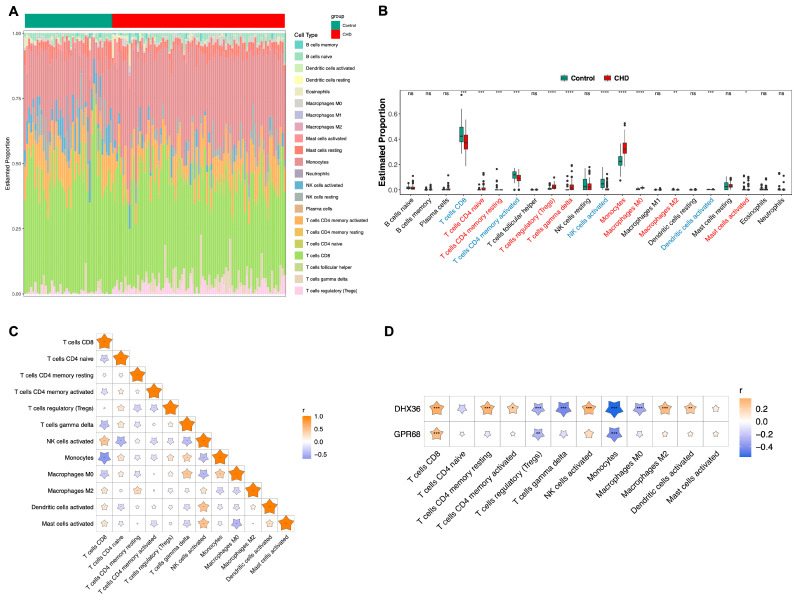
Immune infiltration analysis. (**A**) Stacked bar chart showing the proportion of immune cell infiltration. (**B**) Box plot depicting the immune cell infiltration status, with the *x*-axis representing 22 types of immune cells. Red indicates an increased proportion of immune cells in the CHD group compared to the Control group, while blue indicates a decreased proportion. Significance levels are indicated as follows: ns (not significant); * (*p* < 0.05); ** (*p* < 0.01); *** (*p* < 0.001); **** (*p* < 0.0001). (**C**) Correlation heatmap of immune cells, where orange represents positive correlation and blue represents negative correlation; darker colors indicate stronger correlations. Significance levels are marked as: * (*p* < 0.05); ** (*p* < 0.01); *** (*p* < 0.001). (**D**) Correlation heatmap between biomarkers and differentially infiltrated immune cells, with the *x*-axis representing immune cells and the *y*-axis representing biomarkers. Stars pointing upwards indicate positive correlation, while those pointing downwards indicate negative correlation. Significance levels are as follows: * (*p* < 0.05); ** (*p* < 0.01); *** (*p* < 0.001).

To elucidate the molecular underpinnings and biological pathways in which the identified biomarkers participate, we initiated our investigation by constructing a geneMANIA interaction regulatory network. This network facilitated the identification of significant interaction relationships, predominantly with genes GPR4, MYD88, and IRF7 (Figure 6D). Building upon these findings, we employed single-gene GSEA to further dissect the involvement of these biomarkers in cellular signaling. Our analysis revealed concurrent participation in pathways such as “OLFACTORY TRANSDUCTION,” “NEUROACTIVE LIGAND receptor interaction,” and “ubiquitin-mediated proteolysis” (Figure 6E,F). Additionally, immune infiltration analysis highlighted significant alterations in the cellular abundance of Monocytes, T regulatory cells (Tregs), and CD8+ T cells in the context of CHD. These immune cell populations not only demonstrated substantial changes but also exhibited a high correlation with the biomarkers under investigation, as shown in Figure 7A–D.

**Figure 8:**
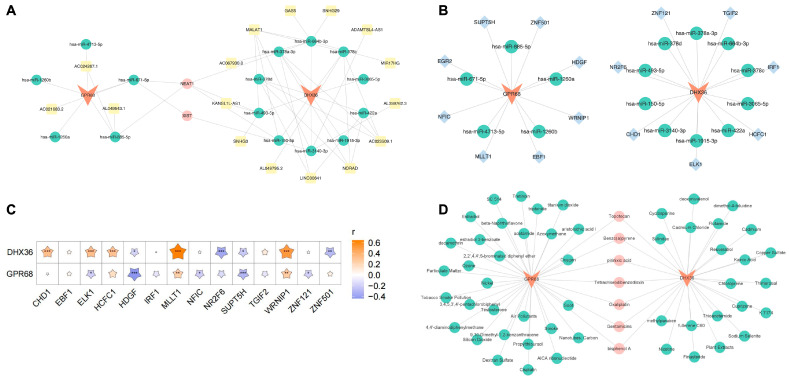
Molecular regulatory network and drug prediction. (**A**) lncRNA-miRNA-biomarker regulatory network, with biomarkers in red, miRNAs in green, lncRNAs in yellow, and lncRNAs associated with two biomarkers in pink. (**B**) TF-biomarker-miRNA regulatory network, with biomarkers in red, miRNAs in green, and TFs in blue. (**C**) Correlation heatmap between biomarkers and TFs, where the *x*-axis represents TFs and the *y*-axis represents biomarkers. Stars pointing upwards indicate positive correlation, while those pointing downwards indicate negative correlation. Significance levels are as follows: * (*p* < 0.05); ** (*p* < 0.01); *** (*p* < 0.001). (**D**) Drug prediction for biomarkers, with biomarkers in red, predicted drugs in green, and co-predicted drugs in pink.

At the non-coding RNA regulatory and transcription factor (TF) level, we identified a suite of molecules associated with the biomarkers, including NEAT1, XIST, KANSL1L-AS1, hsa-miR-671-5p, HDGF, and NR2F6 (Figure 8A–C). In our quest to identify potential therapeutic agents, we discovered several drugs with significant regulatory effects on the biomarkers (Figure 8D). These discoveries may have implications for the development of targeted therapies for CHD.

### 3.6. Experimental Validation of Hub Genes Expression

**Figure 9:**
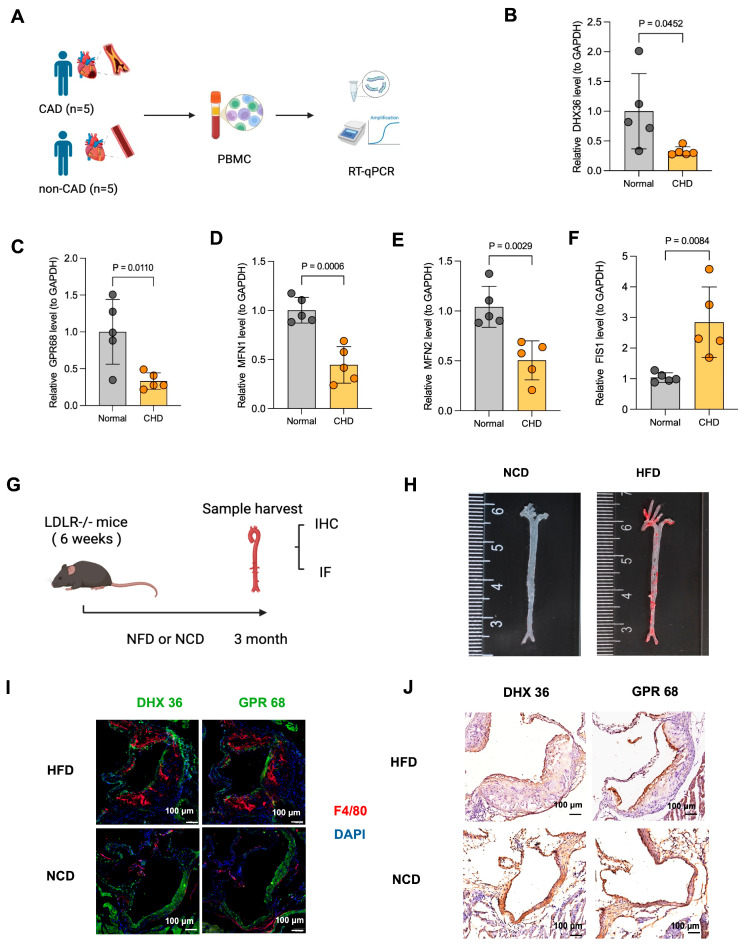
Experimental Validation of hub genes. (**A**) Schematic workflow of qRT-PCR validation using peripheral blood samples from CHD patients and non-CHD controls. Created in BioRender. Junyan, Z. (2026) https://BioRender.com/hbul3u4 (accessed on 28 December 2025). (**B**–**F**) Relative mRNA expression levels of DHX36 (**B**), GPR68 (**C**), MFN1 (**D**), MFN2 (**E**), FIS1 (**F**) in PBMCs from CHD patients (n = 5) compared to non-CHD controls (n = 5). (**G**) Experimental design for establishing the atherosclerosis mouse model using 6-week-old male LDLR−/− mice fed with HFD or NCD for 3 months. Created in BioRender. Junyan, Z. (2026) https://BioRender.com/hbul3u4 (accessed on 28 December 2025). (**H**) Representative images of Oil Red O staining showing atherosclerotic plaque formation in whole-mount aortas. (**I**) Representative immunofluorescence staining images demonstrating the expression of DHX36 and GPR68 (green) in atherosclerotic plaques, with nuclei counterstained with DAPI (blue). (**J**) Representative immunohistochemical staining images showing the expression of DHX36 and GPR68 in atherosclerotic plaque regions.

In the final validation phase, we confirmed the expression patterns of the two hub genes using qRT-PCR (Figure 9A). Peripheral blood was collected from both CHD patients and non-CHD controls, from which peripheral blood mononuclear cells (PBMCs) were isolated for RNA extraction. The results demonstrated that the mRNA expression levels of DHX36 and GPR68 were significantly downregulated in CHD samples compared to controls (*p* < 0.05) (Figure 9B,C). The baseline clinical characteristics of these 10 study participants are presented in Table 2.

Furthermore, to investigate the involvement of MAMs in atherosclerosis pathogenesis, we examined the expression of three core MAMs-related molecules—Mitofusin 1 (MFN1), Mitofusin 2 (MFN2), and Mitochondrial Fission 1 (Fis1)—in the same PBMC samples. Notably, all three markers exhibited significant dysregulation in CHD patients compared to non-CHD controls (*p* < 0.05) (Figure 9D–F). These findings provide direct clinical evidence supporting the critical role of MAMs in the development and progression of atherosclerosis, thereby reinforcing the biological relevance of our identified hub genes, which are functionally associated with MAMs.

To further validate these findings, we established an atherosclerosis mouse model using 6-week-old male LDLR−/− mice fed either a high-fat diet (HFD) or normal chow diet (NCD) for 3 weeks (Figure 9G). Following the dietary intervention, aortas were harvested for gross morphological examination using Oil Red O staining (Figure 9H), which revealed extensive atherosclerotic plaque formation in the HFD group. Both immunofluorescence staining (Figure 9I) and immunohistochemical analysis (Figure 9J) consistently demonstrated downregulation of DHX36 and GPR68 in atherosclerotic plaque regions. These findings corroborate the observations from public databases, suggesting that DHX36 and GPR68 may serve as critical regulatory factors in the pathogenesis of CHD.

## 4. Discussion

This study elucidates the causal relationship between MRGs and CHD through a mendelian randomization approach, thereby identifying potential biomarkers, DHX36 and GPR68, associated with CHD. The exploration of the biological pathways and immune cell involvement linked to these biomarkers not only enhances our understanding of the underlying mechanisms of CHD but also offers novel insights for therapeutic strategies and clinical diagnostics. The findings underscore the importance of MRGs in the pathophysiology of CHD and pave the way for future research aimed at elucidating the intricate interplay between mitochondrial function and cardiovascular health. This work contributes to the growing body of evidence supporting the role of MRGs in CHD, highlighting their potential as targets for intervention and further investigation in the quest for improved patient outcomes.

### 4.1. Role of MAMs in CHD

MAMs are critical membranous contact sites between mitochondria and the endoplasmic reticulum, regulating essential physiological functions such as lipid and Ca^2+^ homeostasis, mitochondrial dynamics, autophagy, and apoptosis. Disruption of ER-mitochondrial communication is a significant contributor to cellular homeostasis alterations, leading to severe diseases, including cancer, neurodegenerative disorders, and cardiovascular diseases. Recent investigations have highlighted MAMs as potential biomarkers and therapeutic targets for cardiovascular pathology [17,18]. However, the specific mechanisms by which MAMs influence CHD remain inadequately understood, necessitating further exploration to identify preventive and therapeutic targets. In this study, we conducted bioinformatics analysis using the GEO database and Mendelian randomization to identify core genes with significant causal relationships with CHD. We then employed PPI algorithms and machine learning techniques to select feature genes, ultimately identifying two biomarkers, DHX36 and GPR68, that exhibited consistent and significant differential expression. By investigating the causal relationship between MRGs and CHD, along with their associated biological pathways and immune cell involvement, this research aims to provide new insights for the treatment and clinical diagnosis of CHD, paving the way for future investigations in this field.

### 4.2. Identification and Characterization of Biomarkers DHX36 and GPR68

DHX36 (DEAH-box helicase 36) is a member of the DEAH-box helicase family and plays a crucial role in RNA metabolism, including RNA unwinding, splicing, and degradation [19]. It is believed to be involved in the regulation of various cellular processes such as gene expression and cellular stress responses [20]. Recent studies have highlighted its potential role in inflammatory and immune responses, which are critical in the pathogenesis of atherosclerosis. Research conducted by Kim et al. has shown that DHX36 is associated with the production of IFN-β and the nuclear translocation of IRF7 induced by CpG-A, playing a role in the viral sensing of plasmacytoid dendritic cells [21]. Additionally, a study by Jing et al. found that DHX36-MyD88 plays a crucial role in recognizing the PRRSV nucleocapsid protein and subsequently activating the pro-inflammatory NF-κB pathway in pigs [22]. On the other hand, GPR68 (G protein-coupled receptor 68), also known as OGR1, is a proton-sensing receptor that plays an important role in cellular responses to changes in pH and is involved in processes like bone remodeling and immune responses. Research indicates that GPR68 functions as a flow sensor in endothelial cells, playing a crucial role in the local regulation of vascular resistance and the flow-mediated dilation process. Impairments in these processes are precursors to vascular diseases such as atherosclerosis [23]. Furthermore, the responsiveness of GPR68 to mechanical and chemical stimuli suggests that it may influence the onset and progression of atherosclerosis through multiple pathways under various pathological conditions [23].

Although no existing studies have directly reported the correlation between DHX36, GPR68 and MAMs, we hypothesize that DHX36 may influence mitochondrial biogenesis and ER stress responses by modulating the stability and translation of mRNAs encoding essential mitochondrial and ER proteins, given its critical role in regulating G-quadruplex structures and maintaining mRNA processing [24,25]. Similarly, GPR68 is primarily involved in the cellular sensing of acidic environments [23]. We hypothesize that its activation may influence mitochondrial energy metabolism and ER function by regulating intracellular pH levels. Furthermore, GPR68 may play a role in cellular stress responses and inflammatory reactions, potentially affecting cell survival and death by modulating the interaction between mitochondrial and ER functions. However, the specific mechanisms underlying these processes require further foundational experiments for elucidation.

### 4.3. Construction of Molecular Regulatory Networks

Simultaneously, we constructed a molecular regulatory network to visualize the interactions of the lncRNA-miRNA and the two selected biomarkers. This network comprises two biomarkers, 15 miRNAs, and 18 lncRNAs, with a total of 77 regulatory relationships identified. Notably, the lncRNAs associated with both biomarkers are NEAT1 and XIST. Previous studies have highlighted the significant roles of NEAT1 and XIST in the progression of atherosclerosis and CHD. NEAT1 regulates inflammatory responses, lipid uptake, oxidative stress, and immune functions through various molecular mechanisms [26,27,28], while XIST primarily modulates apoptosis and injury responses [29]. For instance, Vlachogiannis et al. reported an increased expression of NEAT1 lncRNA in peripheral blood mononuclear cells of patients with coronary artery disease, which is associated with ADAR1-catalyzed A-to-I RNA editing in atherosclerotic cardiovascular disease [30]. Furthermore, Yang et al. demonstrated that exercise improves atherosclerosis by downregulating NEAT1 via N6-methyladenosine modification and METTL14 [31]. Additionally, Xu et al. found that XIST is upregulated in human umbilical vein endothelial cells (HUVECs) stimulated by oxidized low-density lipoprotein (ox-LDL) and the knockdown of XIST can enhance cell viability and inhibit apoptosis by regulating the miR-320/NOD2 axis [29]. These findings provide new potential therapeutic targets for atherosclerosis and CHD.

Also, we identified 15 transcription factors (TFs) linked to 2 biomarkers and constructed a regulatory network involving 15 miRNAs. Pearson correlation analysis showed that biomarker DHX36 had the highest positive correlation with TF MLLT1 (0.6436, *p* < 0.001), while biomarker GPR68 had the highest negative correlation with TF HDGF (−0.4364, *p* < 0.001).

### 4.4. Pathway Enrichment Analysis

#### 4.4.1. Olfactory Transduction Pathway

The enrichment analysis identified several critical pathways associated with the biomarkers DHX36 and GPR68, which may play pivotal roles in the pathogenesis of atherosclerosis. Notably, the olfactory transduction pathway emerged prominently in the analyses of both DHX36 and GPR68. A study by Orecchioni et al. published in *Science* demonstrated that olfactory receptor 2 (OLFR2) plays a role in the pathogenesis and plaque formation of atherosclerosis by activating the NLRP3 inflammasome and inducing the secretion of interleukin-1β [32]. Rayner et al. even referred to this finding as the “*The Scent of Atherosclerosis*” [33]. Furthermore, gene knockout of Olfr2 or the use of its antagonist, such as citral, significantly reduced the formation of atherosclerotic plaques, indicating that olfactory receptors may serve as potential targets for the prevention and treatment of atherosclerosis [34]. The involvement of DHX36 and GPR68 in this pathway could indicate a novel mechanism through which sensory signaling pathways intersect with cardiovascular pathology.

#### 4.4.2. Spliceosome Pathway

Additionally, the spliceosome pathway, which is essential for mRNA processing, was significantly enriched for DHX36. The spliceosome is a complex of proteins and RNA that facilitates the removal of introns from precursor mRNA, a process critical for the production of functional proteins. Research findings indicate that mice lacking the regulation of splicing by the EDA exon of fibronectin (FN) exhibit a 40% reduction in atherosclerotic lesions, which suggests that the regulated splicing of the EDA exon plays a crucial role in the progression of atherosclerosis [35]. Calpain-6 (CAPN6) enhances the phagocytic activity of macrophages by disrupting the exon junction complex (EJC)-driven mRNA splicing, thereby promoting the development of atherosclerosis. Mice deficient in CAPN6 demonstrate decreased lipid deposition in macrophages and reduced atherosclerotic lesions [36]. The association of DHX36 with the spliceosome suggests that it may influence gene expression profiles relevant to atherosclerosis, potentially affecting the stability and function of mRNAs involved in inflammatory responses and lipid metabolism.

#### 4.4.3. Ubiquitin-Mediated Proteolysis Pathway

The ubiquitin-mediated proteolysis pathway, which is enriched in both DHX36 and GPR68, plays a crucial role in protein degradation and turnover, essential for maintaining cellular homeostasis. Ubiquitin-mediated proteolysis is implicated in various pathophysiological states in humans, including inflammatory diseases, neurodegenerative disorders, and muscular dystrophies [37]. This study innovatively proposes the potential role of the ubiquitin-mediated proteolysis pathway in atherosclerosis and CHD through enrichment analysis. Although the specific role of ubiquitin-mediated protein degradation in atherosclerosis is not yet fully understood, the ubiquitin system’s involvement in regulating inflammatory responses may influence the progression of atherosclerosis. Furthermore, under stress conditions such as hypoxia, the ubiquitin system modulates cellular stress responses by degrading specific proteins, which may play a role in the pathological processes of atherosclerosis [37]. Collectively, these pathways underscore the multifaceted roles of DHX36 and GPR68 in atherosclerosis and warrant further investigation into their potential as therapeutic targets.

### 4.5. Immune Infiltration Analysis

Furthermore, our immune infiltration analysis revealed significant differences in the proportions of 12 immune cell types between the Control group and the CHD group. We also identified notable correlations between specific immune cell and the biomarkers DHX36 and GPR68 in the context of CHD. Notably, CD8+ T cells exhibited the highest positive correlation with both DHX36 and GPR68. In recent years, the role of CD8+ T cells in atherosclerosis has garnered increasing attention. Previous studies have suggested that CD8+ T cells display activated and cytotoxic characteristics within atherosclerotic plaques, contributing to macrophage death and necrotic core formation, thereby exacerbating atherosclerosis [38,39]. Research by Cochain et al. further indicated that CD8+ T cells promote atherosclerosis by modulating monocyte generation and circulating monocyte levels, thereby increasing the burden of macrophages in plaques without directly affecting monocyte recruitment [40]. Conversely, the study by Duijn et al. demonstrated that CD8+ T cells can exert a protective role in advanced atherosclerosis by limiting the accumulation of Th1 cells and macrophages [41]. In our analysis, we observed that CD8+ T cell levels were lower in the CHD group compared to the Control group, yet they exhibited a strong positive correlation with the two biomarkers we identified. This leads us to hypothesize that CD8+ T cells may further inhibit the progression of atherosclerosis through mechanisms involving DHX36 or GPR68. However, this hypothesis requires further experimental validation.

Conversely, monocytes demonstrated the highest negative correlation with both DHX36 and GPR68. Monocytes play a pivotal role in the innate immune response, as their differentiation into macrophages is a key event in the pathogenesis of atherosclerosis, contributing to lipid uptake and the formation of foam cells, which are hallmarks of atherosclerotic lesions [42,43,44]. The negative correlation with these biomarkers suggests that higher levels of DHX36 and GPR68 may be associated with reduced monocyte activity or recruitment, which could impact the inflammatory processes underlying atherosclerosis.

Additionally, regulatory T cells (Tregs) exhibited a negative correlation with both DHX36 and GPR68. Previous studies have demonstrated that Tregs and their secreted inhibitory cytokines, such as IL-10 and TGF-β, can play a protective role in atherosclerotic lesions [45]. Furthermore, research by Klingenberg et al. has shown that Tregs can inhibit atherosclerosis by modulating lipoprotein metabolism [46]. Given the important role of Tregs in inhibiting atherosclerosis, enhancing the function or number of Tregs may represent a novel therapeutic strategy.

### 4.6. Development of Diagnostic Model

In this study, we developed a diagnostic model for CHD using two biomarkers associated with MAMs, achieving an impressive AUC value of 0.94. This highlights the potential of DHX36 and GPR68 as excellent diagnostic markers for CHD. Currently, there is a notable lack of effective serum biomarkers for atherosclerosis and CHD, as the diagnosis primarily relies on clinical assessments and imaging techniques [47]. Although serum biomarkers, including lipid levels, inflammatory markers, and homocysteine, may offer additional diagnostic insights into atherosclerosis development, a significant gap remains in the availability of highly accurate and specific biomarkers for the early diagnosis and screening of CHD patients [48,49]. Our findings, derived from bioinformatics analyses, identified two suspicious gene-related biomarkers that could pave the way for more precise diagnostic tools, thereby enhancing early detection and improving patient outcomes in the management of atherosclerosis and CHD.

### 4.7. Limitation

This study is not without its limitations, which warrant careful consideration. Firstly, the absence of corroborative wet lab experiments restricts the validation of our findings, potentially undermining the robustness of the conclusions drawn. Secondly, our study is limited by the small sample size of human PBMCs and the lack of associated clinical data, which restricts the applicability of our findings to real-world scenarios and prevents us from gaining deeper insights into their clinical implications. Furthermore, the utilization of multiple datasets introduces the possibility of batch effects, which may confound the analysis and obscure the true biological signals. Finally, while our experimental validation employed an LDLR−/− atherosclerosis mouse model, this approach was selected based on atherosclerosis being the predominant underlying pathology in CHD, accounting for the majority of cases in our clinical cohort. The LDLR−/− model effectively recapitulates key pathophysiological features relevant to CHD, including endothelial dysfunction, lipid accumulation, and inflammatory responses. However, we acknowledge that this model may not capture all CHD phenotypes, particularly acute thrombotic events or non-atherosclerotic mechanisms. Future studies incorporating additional models, such as myocardial infarction or ischemia–reperfusion models, would provide complementary insights into the broader spectrum of CHD pathology and further validate the roles of DHX36 and GPR68 across different disease contexts. These limitations highlight the necessity for future research to incorporate experimental validation, clinical correlation, and rigorous data normalization techniques to enhance the reliability and translational potential of the results.

## 5. Conclusions

In conclusion, this study presents initial findings that suggest a possible causal relationship between MRGs and CHD through a comprehensive analytical approach. By leveraging transcriptomic sequencing data, Mendelian randomization analysis, and machine learning algorithms, we have identified two potential candidate biomarkers, DHX36 and GPR68, that warrant further investigation. Our exploratory investigation into the biological pathways, immune cell involvement, and molecular mechanisms associated with these biomarkers has begun to enhance our understanding of the underlying pathophysiology of CHD and may indicate potential avenues for therapeutic strategies and clinical diagnostics. Importantly, the specific mechanisms and molecular targets identified in this study should be considered preliminary and require rigorous validation in larger, independent cohorts and diverse populations to establish their roles in the development and progression of CHD. This research serves as a starting point for future studies to explore the intricate interplay between mitochondrial function and cardiovascular health, with the ultimate aspiration of improving patient outcomes through targeted interventions and a better understanding of the genetic basis of CHD.

## Figures and Tables

**Table 1:** Primer sequence for qRT-PCR.

Genes	Primer Sequence (5′–3′)
*DHX36*	F: GCTGGTTCTGACGGGTTGTA
*DHX36*	R: GTACCACATGCCGATTTCGC
*GPR68*	F: GTGGAATCGGAGCACAACT
*GPR68*	R: TCCCAGTCTCTGACTCACCT
*GAPDH*	F: CGAAGGTGGAGTCAACGGATTT
*GAPDH*	R: ATGGGTGGAATCATATTGGAAC

**Table 2:** Baseline characteristics of included patients.

	Control 1	Control 2	Control 3	Control 4	Control 5	CHD 1	CHD 2	CHD 3	CHD 4	CHD 5
Age	75	87	65	73	75	71	77	78	81	75
Sex	Male	Male	Female	Female	Male	Female	Female	Male	Male	Male
Hypertension	Yes	No	No	No	Yes	Yes	Yes	Yes	No	No
Diabetes	No	No	No	No	Yes	Yes	Yes	No	No	No
Smoking	No	No	No	No	Yes	No	No	No	No	Yes
CHD subtype	/	/	/	/	/	CCS	CCS	UA	CCS	CCS

Abbreviations: CHD, Coronary Heart Disease; CCS, Chronic Coronary Syndrome; UA, Unstable Angina.

## Data Availability

The original contributions presented in this study are included in the article/Supplementary Material. Further inquiries can be directed to the corresponding author.

## Supplementary Materials

The following supporting information can be downloaded at https://www.mdpi.com/article/10.3390/cimb48010075/s1.

## Abbreviations

The following abbreviations are used in this manuscript:

AUC	Area Under the Curve
BP	Biological Process
CC	Cellular Component
CHD	Coronary Heart Disease
DEGs	Differentially Expressed Genes
DE-MRGs	Differentially Expressed MAM-Related Genes
ER	Endoplasmic Reticulum
GEO	Gene Expression Omnibus
GO	Gene Ontology
GSEA	Gene Set Enrichment Analysis
GWAS	Genome-Wide Association Study
HFD	High-Fat Diet
IVW	Inverse Variance Weighted
KEGG	Kyoto Encyclopedia of Genes and Genomes
LASSO	Least Absolute Shrinkage and Selection Operator
LDLR	Low-Density Lipoprotein Receptor
lncRNA	Long Non-Coding RNA
log2FC	Log2 Fold Change
MAMs	Mitochondria-Associated Endoplasmic Reticulum Membranes
MF	Molecular Function
miRNA	MicroRNA
MRGs	MAM-Related Genes
mRNA	Messenger RNA
ox-LDL	Oxidized Low-Density Lipoprotein
PBMCs	Peripheral Blood Mononuclear Cells
PPI	Protein–Protein Interaction
qRT-PCR	Quantitative Real-Time Polymerase Chain Reaction
ROC	Receiver Operating Characteristic
SNPs	Single Nucleotide Polymorphisms
ssGSEA	Single-Sample Gene Set Enrichment Analysis
SVM-RFE	Support Vector Machine-Recursive Feature Elimination
TFs	Transcription Factors
WGCNA	Weighted Gene Co-Expression Network Analysis
