# Supplementary material for: Mitochondria-Associated Endoplasmic Reticulum Membrane Biomarkers in Coronary Heart Disease and Atherosclerosis: A Transcriptomic and Mendelian Randomization Study
Source: Curr Issues Mol Biol. 2026 Jan 12;48(1):75. doi: 10.3390/cimb48010075 (PMC12840513; doi:10.3390/cimb48010075)
Supplement: Supplementary file 1 [file cimb-48-00075-s001.zip › cimb-4055117-supplementary.pdf]

Supplementary Table

Table S1 MR analysis on significant causal exposure factors and CHD.

Symbol	exposure	outcome	method	nsnp	pval	OR	OR_lci95	OR_uci95
GPR68	eqtl-a-ENSG00000119714	Major coronary heart disease event || id:ukb-d-I9_CHD	Inverse variance weighted (multiplicative random effects)	3	0.043288017	0.997035005	0.994167788	0.999910492
DHX36	eqtl-a-ENSG00000174953	Major coronary heart disease event || id:ukb-d-I9_CHD	Inverse variance weighted (multiplicative random effects)	3	2.08E-27	0.997240344	0.996742462	0.997738475

OR value, 1 as the boundary, greater than 1 as risk factor, less than 1 as safety factor; Abbreviations: MR, Mendelian randomization; SNP ,Single nucleotide polymorphism; CHD,Coronary heart disease.

Table S2. MR heterogeneity test and analysis.

Symbol	exposure	outcome	method	Q	Q_df	Q_pval
GPR68	eqtl-a-ENSG00000119714	Major coronary heart disease event || id:ukb-d-I9_CHD	Inverse variance weighted	2.749109729	2	0.252952169
DHX36	eqtl-a-ENSG00000174953	Major coronary heart disease event || id:ukb-d-I9_CHD	Inverse variance weighted	0.186636876	2	0.910903388

Q_pval value greater than 0.05 indicates that there is no heterogeneity among the samples.

Table S3. The horizontal pleiotropy test.

Symbol	id.exposure	outcome	exposure	egger_intercept	se	pval
GPR68	eqtl-a-ENSG00000119714	Major coronary heart disease event || id:ukb-d-I9_CHD	ENSG00000119714 || id:eqtl-a-ENSG00000119714	0.000383942	0.000845777	0.728713818
DHX36	eqtl-a-ENSG00000174953	Major coronary heart disease event || id:ukb-d-I9_CHD	ENSG00000174953 || id:eqtl-a-ENSG00000174953	-0.000144868	0.000565624	0.840379532

p>0.05 indicates that there was no horizontal pleiotropy.CHD,coronary heart disease.
